# Development of Fluorine Fixation Processes for the Horizontal Recycling of Lithium

**DOI:** 10.3390/ma18092050

**Published:** 2025-04-30

**Authors:** Kazuki Fujiwara, Kaisei Ito, Shunsuke Kuzuhara, Osamu Terakado, Natsuki Hosoya, Hideo Hayashi, Ryo Kasuya

**Affiliations:** 1 National Institute of Technology, Sendai College, 48 Nodayama Medeshima-Shiote, Miyagi, Natori 981-1239, Japan; fujiwara.kazuki.q7@dc.tohoku.ac.jp (K.F.);; 2 National Institute of Technology, Hakodate College, Tokura-cho 14-1, Hokkaido, Hakodate 042-8501, Japan; terakado@hakodate-ct.ac.jp; 3 Yamaguchi Prefectural Industrial Technology Institute, 4-1-1, Asutopia, Ube, Yamaguchi 755-0195, Japan; hosoya@iti-yamaguchi.or.jp; 4 Tokyo Metropolitan Industrial Technology Research Institute, 2-4-10, Aomi, Koto-ku, Tokyo 135-0064, Japan; hayashi.hideo@iri-tokyo.jp; 5 National Institute of Advanced Industrial Science and Technology (AIST), 16-1 Onogawa, Ibaraki, Tsukuba 305-8569, Japan; ryo-kasuya@aist.go.jp

**Keywords:** lithium-ion battery, lithium carbonate, fluoride fixation, calcium fluoride, hydrotalcite

## Abstract

In order to effectively recover Li from cathode active materials of lithium-ion batteries, model samples of LiCoO_2_ mixed with polyvinylidene fluoride (PVDF) were calcined at temperatures of 350–700 °C under an Ar or air atmosphere. Complete Li recovery was achieved by calcining the model sample at 400 °C under an Ar atmosphere, followed by water leaching. Additionally, to immobilize PVDF-derived F, an impurity in Li purification, we explored the use of calcium compounds (Ca(OH)_2_ and CaCO_3_) and a layered double hydroxide in both dry and wet processing methods. In the wet process, F was fixed by adding Ca(OH)_2_ to an aqueous LiF solution containing 1000 ppm of F. We confirmed that 98.6% of F was successfully removed from the solution after repeated fixation procedures. Furthermore, the unreacted Ca in the solution was separated and removed as CaCO_3_ by concentrating the solution.

## 1. Introduction

Lithium-ion batteries (LIBs) are key devices in the transition to electric vehicles owing to their high energy densities and long cycle lives. In 2020, 21.6 tons of Li and 80.9 tons of Ni were used for electric vehicles and stationary energy storage systems, respectively. According to the sustainable development scenario proposed by the International Energy Association, the demand for these metals will increase approximately 40-fold by 2040, compared with that in 2020. Similarly, the demand for Co and Mn (21.3 and 25.6 tons, respectively, in 2020) will increase 21- and 16-fold, respectively [1]. In light of these rapid increases in metal demand, the European Union recently enacted two new regulations: the Battery Regulation [2] and the Critical Raw Materials Act [3]. The Battery Regulation mandates the recovery of metals from rechargeable batteries with a capacity of ≥2 kWh and the inclusion of a certain amount of recovered metals (Li, Co, and Ni). For Li, 80% is the target recovery rate by the end of 2031. Moreover, after August 2036, at least 12% of recovered Li must be included in the active materials. To comply with this regulation, the metals in spent batteries must be refined to battery-grade chemicals. For example, Li carbonate (Li_2_CO_3_) with a purity of ≥99.5% is traded as battery grade.

The Critical Raw Materials Act aims to lower the dependence on third-world countries or regions for substances defined as strategic raw materials below a certain level. For this purpose, the following benchmarks for the annual consumption of strategic raw materials in the European region have been set, and these values must be approached or achieved: ore mining capacity (≥10%), processing and other intermediate treatment capacities (≥40%), and recycling capacity (≥15%). LIBs mainly include the following strategic raw materials: Li, Ni, Co, and Mn as cathode active materials (CAMs), Al and Cu as current collectors, and graphite as the anode active material. In addition, the Japanese government announced a plan to mandate the recycling of LIB process scraps [4]. Therefore, closed-loop recycling has become increasingly important.

Various methods have been proposed for metals recovery from spent LIBs, including dry and wet processes and recent direct recycling [5,6,7,8,9]. The pyrometallurgical process is based on smelting technologies, and it enables the facile recovery of Co, Ni, and Cu. For example, Umicore recovers metals from spent LIBs in a high-temperature (1200–1450 °C) process using coke as the reducing agent [10]. Co, Ni, and Cu melt at high temperatures to form alloys, whereas Li, Mn, and Al form slag (oxide) phases, the byproducts of which can be used in construction processes (e.g., cement) [10]. However, because the Li concentration in slag is very low, there is a high economic hurdle in achieving closed-loop recycling.

Previously, we successfully separated and recovered Li and Co from a CAM model (LiCoO_2_) via carbothermal reduction using activated carbon as the reducing agent [11]. The reaction between LiCoO_2_ and carbon powder proceeds as follows at 500 °C in an inert atmosphere (Equation (1)):

2LiCoO_2_ + C → Li_2_CO_3_ + CoO + Co (1)

Li_2_CO_3_ is soluble in water, whereas the other products are not. Therefore, Li and Co can be separated via water leaching. A combination of thermal treatment and subsequent leaching is one of the most important strategies for recovering elements from spent LIBs [12,13]. This “acid-free” process has considerable environmental and cost advantages over conventional hydrometallurgical [14,15] and hydrothermal processes [16], which use various types of acids to extract Li from spent LIBs.

The application of this process to spent cathode materials (containing LiCoO_2_) collected from laptop PCs demonstrated that Li could be leached without the addition of activated carbon [17]. Because the trivalent Co in LiCoO_2_ is reduced to divalent and zerovalent, it is assumed that the carbon contents of the conductive additive (e.g., acetylene black) and binder (e.g., polyvinylidene fluoride, PVDF) act as reducing agents. In addition, the leachate contained not only Li but also F. This indicates that fluorine derived from PVDF and the electrolyte (lithium hexafluorophosphate, LiPF_6_) were eluted into the water.

When Li salt is precipitated and recovered through evaporation and drying of the leachate, F contamination reduces its purity. Based on the results obtained with the model solutions, the F/Li ratio (mass ratio) in the leachate must not exceed 0.05 to achieve 99% purity of Li_2_CO_3_ [11]. However, the F/Li ratio of the leachate obtained by processing spent LIBs is much higher, at 0.8 [16]. Therefore, we investigated the immobilization of fluorine by adding Ca compounds to the leachate [14]. Under optimal conditions, 98% of F in the leachate was fixed as Ca fluoride (CaF_2_). However, there was an issue with Ca remaining in the residual solution.

In this study, we investigated the use of Ca compounds and adsorbents to achieve high levels of F fixation. In addition to the types of Ca compounds and approaches used in the dry and wet processes, we also experimented with a multistep process using a combination of Ca compounds and adsorbents. Various materials, including metal-, natural mineral-, and carbon-based materials, have been proposed as F adsorbents [18]. Herein, we chose hydrotalcite [19], which is a type of layered double hydroxide (LDH). This material retains its adsorption properties in solutions with high pH values.

## 2. Materials and Methods

### 2.1. Lithium Recovery and Fluorine Fixation by Dry Process

**Figure 1:**
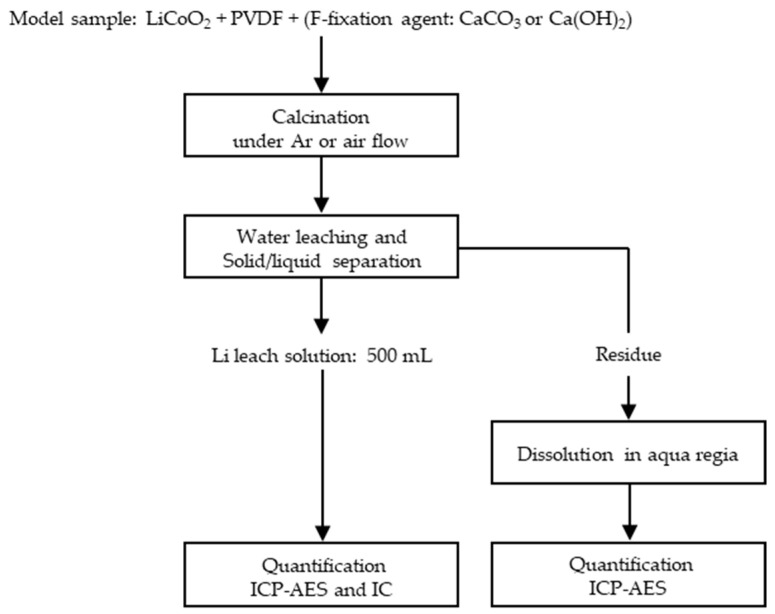
Flowchart of Li recovery and F fixation via the dry process.

Figure 1 shows a flowchart of the drying process. Model samples consisting of LiCoO_2_ powder and PVDF were calcined under an Ar or air atmosphere. The calcined samples were dispersed in water, sonicated, and filtered to obtain the leaching solution. An elemental concentration analysis of the leaching solution was performed to evaluate the leaching efficiencies of Li and F. Ca(OH)_2_ or CaCO_3_ was used as the F-fixing agent and added to the model samples, which were then calcined. In addition to the reaction whereby LiCoO_2_ is reduced by the pyrolysis product of PVDF, as shown in Equation (2), we propose a reaction in which the simultaneously produced HF is inhibited by a Ca compound (Equations (3) and (4)).

2LiCoO_2_ + C(from PVDF) → Li_2_CO_3_ + CoO + Co (2)

Ca(OH)_2_ + 2HF(from PVDF) → CaF_2_ + 2H_2_O (3)

CaCO_3_ + 2HF(from PVDF) → CaF_2_ + CO_2_ + H_2_O (4)

#### 2.1.1. Sample Preparation

LiCoO_2_ powder (Toshima Manufacturing Co. Ltd., Saitama, Japan, 3N; LiLBPW01) was used as the CAM model. A model cathode material sample was prepared by mixing 4.5 g of LiCoO_2_ powder and 0.5 g of PVDF (Sigma-Aldrich Co., St. Louis, MO, USA; 182702) in an agate mortar. In addition, we attempted to fix F by adding Ca(OH)_2_ (FUJIFILM Wako Pure Chemical Co., Osaka, Japan; 038-14171) or CaCO_3_ (FUJIFILM Wako Pure Chemical Co., Osaka, Japan; 035-12322) to a model sample (0.9 g of LiCoO_2_ powder and 0.1 g of PVDF). The atomic ratio of Ca to F was set in the range of 0.25 to 1.5.

#### 2.1.2. Calcination

**Figure 2:**
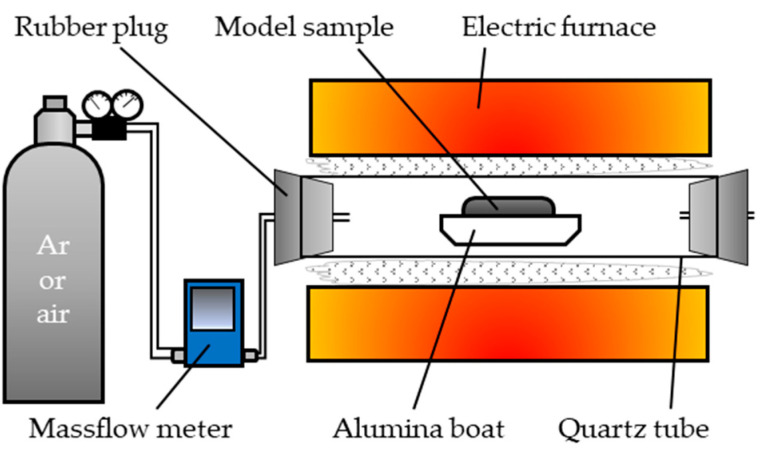
Schematic of the calcination apparatus.

The sample was spread on an alumina boat (SANSYO Corporation, Tokyo, Japan, SAB-995 AB-6) and inserted into the center of a quartz tube (Figure 2) in an electric furnace (Koyo Thermo Systems Corporation, Nara, Japan; KTF030N1). The atmosphere gas of Ar or air was allowed to flow at a rate of 100 mL/min. The temperature was increased in two steps, from room temperature to 100 °C and from 100 °C to the calcination temperature (350–700 °C), both at a rate of 10 °C/min. The holding times were 10 min at 100 °C and 60 min at the calcination temperature. After holding, the sample was cooled to <100 °C, and the calcined sample was crushed in an agate mortar.

#### 2.1.3. Water Leaching

Ultrapure water (250 mL)—50 mL in the case of F fixation via the dry process—was poured into the crushed calcined sample, followed by sonication at 38 kHz for 60 min in an ultrasonic washing machine (SND Co., Nagano, Japan; US-1). The obtained dispersion was filtered under reduced pressure using a polytetrafluoroethylene (PTFE) membrane filter (Toyo Roshi Kaisha, Ltd., Tokyo, Japan; H020A047A) with a pore size of 0.2 µm to separate it into filtrate and residue. The filtrate volume was adjusted to 500 mL—100 mL in the case of F fixation via the dry process—by adding ultrapure water. The residue was dried overnight on a hotplate at a setting temperature of 100 °C, after which the water leaching was repeated under identical conditions.

#### 2.1.4. Dissolution of Water Leaching Residue

The residue of 1.0 g was added to 30 mL of aqua regia and kept at 150 °C for 60 min using a hot plate. Subsequently, the solution was filtered through a reduced-pressure PTFE membrane filter with a pore diameter of 0.2 µm. The filtrate volume was adjusted to 100 mL with ultrapure water.

### 2.2. Fluorine Fixation Via the Wet Process

**Figure 3:**
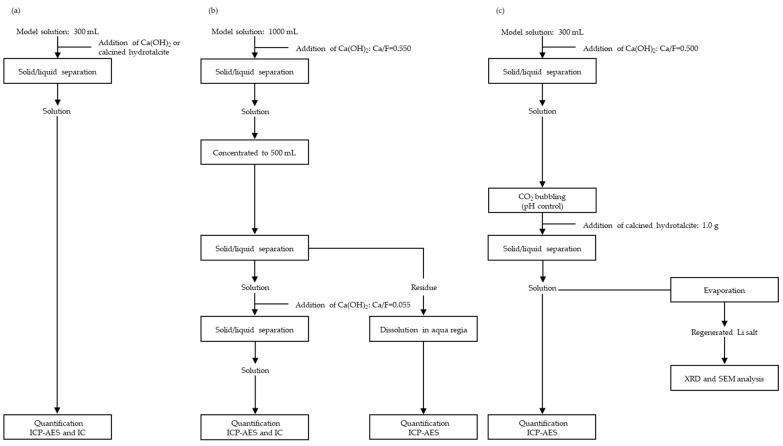
Flowchart of F fixation tests. (**a**) Single step using Ca(OH)_2_ and hydrotalcite, (**b**) multiple steps using Ca(OH)_2_, and (**c**) multiple steps using Ca(OH)_2_ and hydrotalcite.

Figure 3 shows a flowchart of F fixation using the wet process. A LiF model solution with F concentrations ranging from 25 to 1000 ppm was prepared using a LiF reagent (FUJIFILM Wako Pure Chemical Co., Osaka, Japan, Wako Special Grade; 127-01785). Ca(OH)_2_ (FUJIFILM Wako Pure Chemical Co., Osaka, Japan; 038-14171) or hydrotalcite (Mg_6_Al_2_(OH)_16_CO_3_·4H_2_O) (FUJIFILM Wako Pure Chemical Co., Osaka, Japan; 320-87432) was used to evaluate the F fixation capacity (Figure 3a). Before use, the hydrotalcite was calcined at 500 °C for 60 min in an Ar atmosphere.

#### 2.2.1. Single Step

In 300 mL of LiF model solution, Ca(OH)_2_ or calcined hydrotalcite was added at room temperature. The dispersion was stirred at 500 rpm for 120 min for Ca(OH)_2_, and kept for 180 min for hydrotalcite. Here, we assumed that Ca(OH)_2_ was converted into CaF_2_ (Equation (5)).

2F^−^ + Ca^2+^ → CaF_2_ (5)

Therefore, we set the atomic ratio of Ca to F in the solution (Ca/F) from 0.500 to 0.575. Conversely, when adding calcined hydrotalcite, 1.0 g was added to the solution—the expected F adsorption is 88.7 mg-F/g-hydrotalcite [20]. The dispersion was sampled with a syringe (Terumo Corporation, Tokyo, Japan, SS-10ESZ), filtered using a PTFE syringe filter (RephiLe Bioscience, Ltd., Boston, MA, USA; RJF3245NH) with a pore size of 0.45 µm, and then analyzed. The mother solution was filtered under reduced pressure using a PTFE membrane filter with a pore size of 0.2 µm.

#### 2.2.2. Multiple Steps

As shown in Figure 3b,c, multiple steps were applied to the LiF model solution with a F concentration of 1000 ppm. In the case of repeated addition of Ca(OH)_2_ (Figure 3b), the amount of Ca(OH)_2_ added in the primary step was set to achieve a Ca/F atomic ratio of 0.550 based on the F content in 1000 mL of the solution. After the primary fixation of F, the obtained dispersion was filtered and concentrated through evaporation to a solution volume of 500 mL. In the secondary step, Ca(OH)_2_ was added to the concentrated solution at one-tenth of the amount used for primary processing. When Ca(OH)_2_ and calcined hydrotalcite were added (Figure 3c), the amount of Ca(OH)_2_ was adjusted to achieve a Ca/F ratio of 0.500 based on the F content in 300 mL of the solution. After primary processing, the solution was filtered and the pH was adjusted by using CO_2_ bubbling. During secondary processing, calcined hydrotalcite (1.0 g) was added to the solution, and Ar gas was applied at a flow rate of 100 mL/min. After the reaction, the solution was filtered and evaporated to obtain the regenerated Li salt.

### 2.3. Characterization

#### 2.3.1. Elemental Analysis

After dilution of the solution samples by appropriate factors, the concentrations of the metals (Li and Ca) in the leachate and solution were determined by inductively coupled plasma-atomic emission spectrometry (ICP-AES, SPECTRO Analytical Instruments, Kleve, Germany; SPECTRO ARCOS). F concentrations were quantified using ion chromatography (IC, Thermo Fisher Scientific, Inc., Commonwealth, MA, USA; Dionex™ Aquion™ Ion Chromatography System, 22176-60002).

#### 2.3.2. XRD Analysis

The crystalline phase of the regenerated Li salt was identified by powder X-ray diffraction (XRD) analysis (Rigaku Holdings Corporation, Tokyo, Japan; Rigaku SmartLab 9 kW) equipped with CuKα radiation. Measurements were taken at a voltage of 45 kV, a current of 200 mA, and 2*θ* range of 10–80° with a step width of 0.02° and a scanning speed of 20°/min.

#### 2.3.3. SEM Analysis

Field emission scanning electron microscopy (FE-SEM, Hitachi High-Tech Corporation, Tokyo, Japan; SU-8600) and energy-dispersive X-ray spectroscopy (EDS) (Oxford Instruments, Abingdon-on-Thames, UK, Ultim Extreme) were used to analyze the regenerated Li salt. The accelerating voltage was 7.0 kV for Ultim Extreme.

#### 2.3.4. Calculation

The Li and F leaching efficiencies, F fixation rate, and reaction rate of hydrotalcite were calculated based on the ICP-AES and IC results. The leaching efficiency and F fixation rate were calculated using Equation (6) and Equation (7), respectively.

(6) $$\mathrm{Leaching}\;\mathrm{efficiency}\;\left[\%\right]=\frac{\mathrm{Leaching}\;\mathrm{amount}}{\mathrm{Addition}\;\mathrm{amount}\;\left(\mathrm{Model}\;\mathrm{sample}\right)}\times 100$$

(7) $$F\;\mathrm{fixation}\;\mathrm{rate}\;\left[\%\right]=\frac{\mathrm{Addition}\;\mathrm{amount}\;\left(\mathrm{solution}\right)-F\;\mathrm{concentration}\;\left(\mathrm{IC}\right)}{\mathrm{Addition}\;\mathrm{amount}\;\left(\mathrm{solution}\right)}\times 100$$

## 3. Results

### 3.1. Li Recovery Via the Dry Process

#### 3.1.1. Leaching Behavior of Li

**Figure 4:**
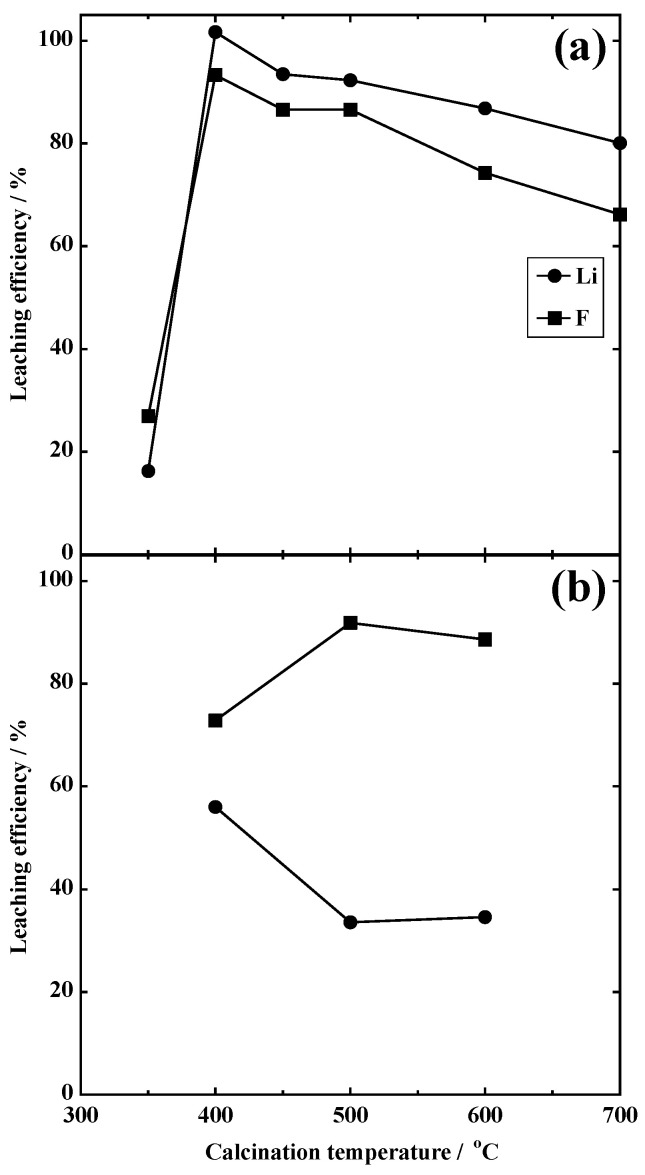
Leaching efficiency of model samples calcined at different calcination conditions. (**a**) Calcined in Ar and (**b**) calcined in air.

Figure 4 shows the relationship between the calcination temperature and the leaching efficiencies of Li and F. Under an Ar atmosphere, the leaching rates at 350 °C were 16.2% for Li and 26.9% for F. Conversely, all of the Li could be leached out, and F showed a maximum value (93.3%) at 400 °C. In the case of spent LIBs, the calcination temperature at which the most Li leached was 500 °C [17]. Spent LIBs contain various components, including Al current collectors and electrolytes primarily composed of LiPF_6_. These constituents significantly influenced both the Li leaching efficiency and the optimal calcination temperature. Therefore, to make this process more practical for Li recovery via dry methods, it is essential to clarify the effects of these materials on the overall process. When the calcination temperature increased over 400 °C, the leaching efficiency of Li and F decreased. At 700 °C, the leaching efficiencies of Li and F were 80.1 and 66.2%, respectively. In contrast, under an air atmosphere, the Li leaching efficiency was much lower than that under an Ar atmosphere, with a maximum of 56.0% at 500 °C. The F leaching efficiency was the highest (91.9%), a different behavior from that under an Ar atmosphere. PVDF combusts in an oxidizing atmosphere [21]; thus, the level of carbon, which acts as a reductant for LiCoO_2_, decreases. Consequently, the reduction in LiCoO_2_ was suppressed, and the Li leaching rate decreased.

Table 1 lists the Li/F ratios of the leaching solutions obtained under different calcination conditions. Under an Ar atmosphere, the Li/F ratio is >3.0 at calcination temperatures over 400 °C. This indicates that the halogenation of LiCoO_2_ and the carbon reduction reaction proceeded simultaneously. Conversely, under the air atmosphere, the value was 2.3 at 400 °C, decreasing to 1.1 at 500 and 600 °C. A Li/F ratio of around 1 suggests that leaching occurred in the form of LiF. These results indicate that an Ar atmosphere is more suitable for Li leaching than under an air atmosphere.

#### 3.1.2. Material Balance of Li

**Figure 5:**
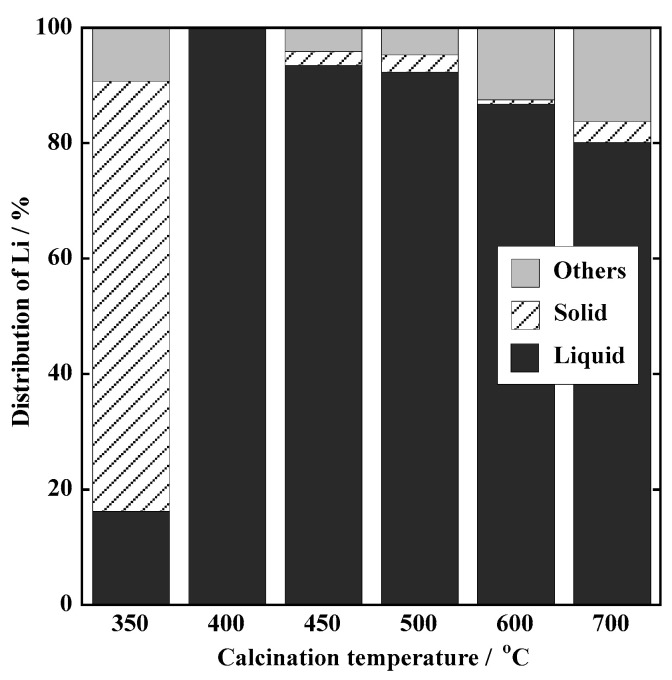
Li distribution in the model samples calcined under Ar at different temperatures.

Under an Ar atmosphere, when the calcination temperature increased over 400 °C, the leaching efficiency of Li decreased. Thus, the material balance of Li was investigated. Figure 5 shows the relationship between the calcination temperature and material balance of Li for the model sample in an Ar atmosphere. At a calcination temperature of 350 °C, water-soluble Li was 16.2% and insoluble Li (solid) was 74.5%. This is because the reduction of LiCoO_2_ did not proceed due to the low calcination temperature. At over 400 °C, water-soluble Li accounts for the majority. The proportion of Li in the solid residue ranged from 0.7 to 3.6% with no difference depending on the calcination temperature. As the calcination temperature increased, Li was distributed to others (4.0–16.4%). Only the sample calcined at 350 °C produced residue when dissolved in aqua regia. Therefore, we can conclude that calcination at high temperatures causes the volatilization of Li, resulting in the loss of Li.

### 3.2. F Fixation for Li_2_CO_3_ Purification

Herein, we developed an efficient method to recover Li by calcination and water leaching; however, PVDF-derived F contamination remains an issue. From the perspective of horizontal recycling of LIBs, the purity of regenerated Li_2_CO_3_ should be battery grade (>99.5%), with a recovery rate over 80%. The F/Li ratio (mass%/mass%) in the leaching solution must be <0.05 to achieve a regenerated Li_2_CO_3_ purity of 99% or higher. The F/Li ratio was 0.87 under the optimum conditions with 100% Li leaching. To obtain high-purity Li_2_CO_3_ with a high recovery rate, it is necessary to suppress LiF formation or fix F in the leachate. Therefore, we investigated F fixation by adding Ca compounds to the model samples during calcination procedure. Furthermore, a combination of Ca compounds and hydrotalcite was applied during the wet process.

#### 3.2.1. Dry Process

**Figure 6:**
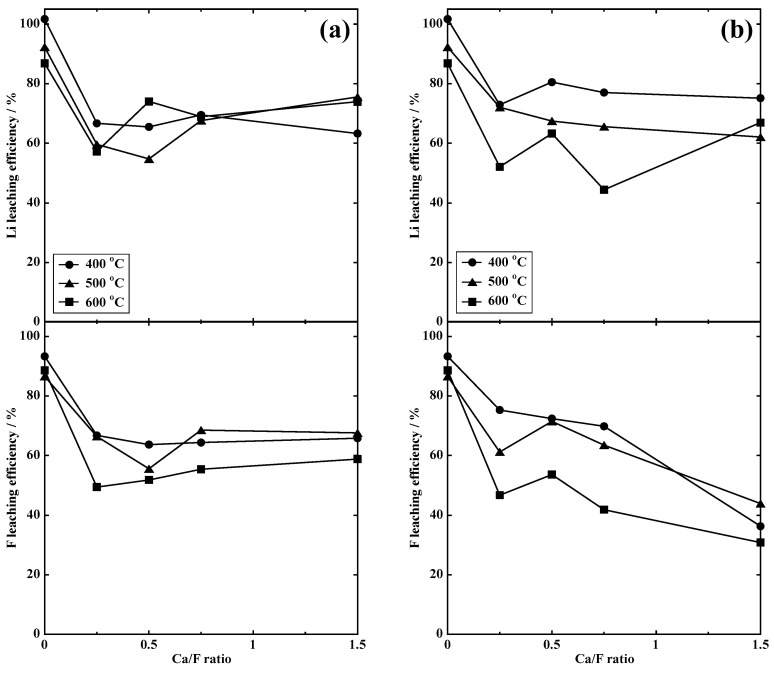
Leaching efficiency of model samples calcined with Ca salts at different temperatures. (**a**) CaCO_3_ and (**b**) Ca(OH)_2_.

Figure 6 shows the relationship between calcination temperature and the leaching efficiencies of Li and F. The leaching of F was successfully suppressed by the addition of Ca compounds. This is due to the formation of sparingly soluble CaF_2_. We noticed that Li leaching was also suppressed at the same time. The sample containing Ca(OH)_2_ (Ca/F = 1.5) exhibited the lowest F leaching efficiency at each calcination temperatures. At 400 °C, the largest difference was observed between Li and F leaching efficiencies; 75.1% for Li and 36.3% for F. Since a target recovery rate (>80%) was not be achieved in these experiments, we attempted the F immobilization in wet conditions.

#### 3.2.2. Wet Process

**Figure 7:**
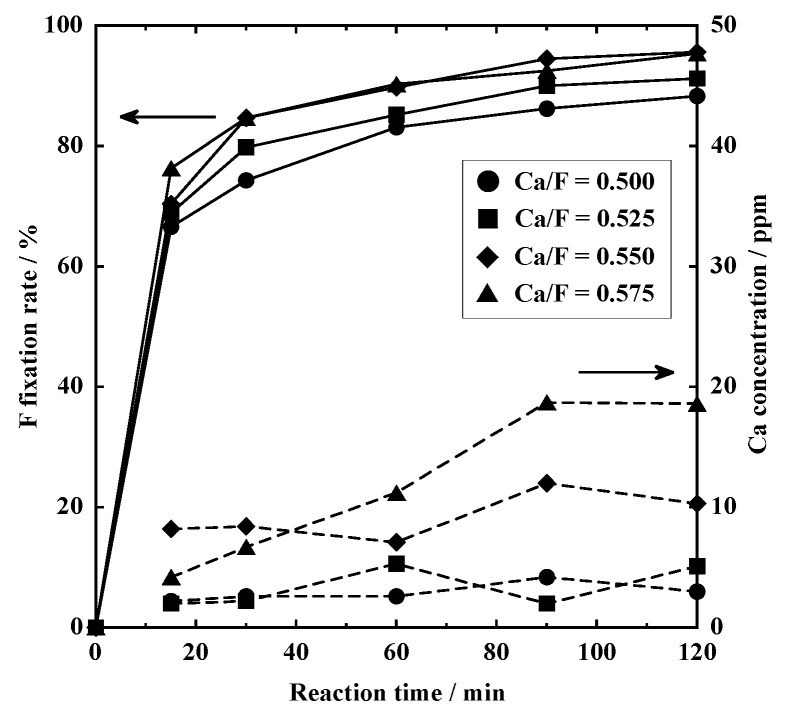
F fixation rate and Ca concentration after the addition of Ca(OH)_2_. Solid lines: F fixation rate; dashed lines: Ca concentration.

Figure 7 shows the changes in the F fixation rate and Ca concentration with the addition of Ca(OH)_2_. The F fixation rate increased rapidly from 66.6 to 76.3% after 15 min, followed by a gradual increase. After 120 min, it reached 88.3% at Ca/F = 0.500, 91.2% at Ca/F = 0.525, 95.6% at Ca/F = 0.550, and 95.4% at Ca/F = 0.575. The residual concentration of Ca was also kept at low levels: 3.0, 5.1, 10.3, and 18.6 ppm at Ca/F = 0.500, 0.525, 0.550, and 0.575, respectively. It should be noted that residual Ca concentrations were much lower than those in our previous study [17].

**Figure 8:**
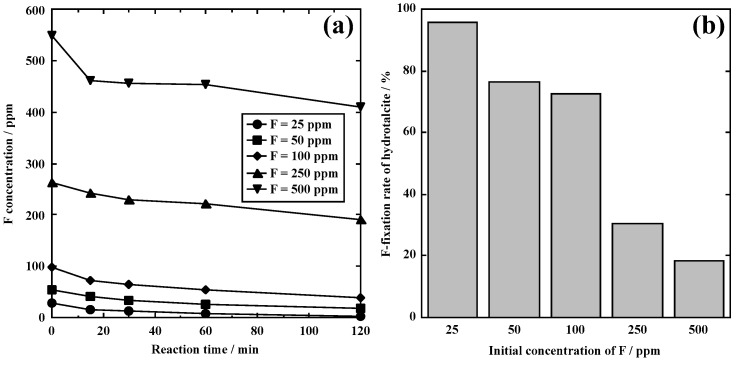
F concentration changes and reaction rate of hydrotalcite. (**a**) F concentration and (**b**) reaction rate of hydrotalcite.

Figure 8 shows the concentration changes in F in the solution after the addition of hydrotalcite and F fixation rate. After 120 min, the F concentration in the solution was 408.3, 173.6, 27.2, 11.8, and 1.0 ppm at F = 500, 250, 100, 50, and 25 ppm, respectively. The F fixation rate of hydrotalcite increased with decreasing initial F concentration; 18.3% at F = 500, 30.6% at F =250, 72.8% at F = 100, 76.4% at F = 50, and 96.0% at F = 25. Notably, the F concentration was lower than the solubility of CaF_2_ (8 ppm) at F = 25. During leaching, the undesired dissolution of metals such as Mg or Al did not occur. From these results, the addition of hydrotalcite is useful when F concentration is low. Effective F fixation will be achieved by adding Ca(OH)_2_ to a solution with a high F concentration, followed by the addition of hydrotalcite.

#### 3.2.3. F Fixed by Secondary Processing

Table 2 lists the F fixation rate and Ca concentration when Ca(OH)_2_ was added to the LiF solution. During primary and secondary processing, 98.2% and 7.4% of the remaining F were fixed, respectively, resulting in a total F fixation rate of 98.6%. The Ca concentration was 57.4 ppm at the end of the primary processing but was drastically reduced to 1.1 ppm by the concentration operation. The decrease in Ca concentration is attributable to the precipitation of Ca-containing compounds. The Ca concentration of the precipitate was measured and calculated by weight as CaCO_3_. The obtained results were balanced against the precipitate weights. This indicated that residual Ca could be removed by concentrating the solution. In addition, a F/Li ratio (mass ratio) of 0.04 was achieved. However, the secondary process resulted in an increase in Ca concentration. The F fixation effect of multiple Ca(OH)_2_ additions is small and would lead to a decrease in Li_2_CO_3_ purity. Additionally, stirring in air causes CO_2_ dissolution into the aqueous solution; thus, an inert gas distribution is necessary to avoid the carbonation of Ca(OH)_2_.

Table 3 summarizes the F fixation rate, Ca concentration, and pH change when Ca(OH)_2_ was added as the primary treatment and hydrotalcite as the secondary treatment. During primary and secondary processing, 89.1% and 44.0% of the remaining F were fixed, respectively, resulting in a total F fixation rate of 95.8%. The F concentration was 109.5 ppm with the addition of Ca(OH)_2_ and 61.3 ppm with the addition of hydrotalcite. We noticed that the F fixation rate of hydrotalcite in the secondary process was much lower than that in the single step (69.4%), despite the F concentration being ~100 ppm. This was due to the preferential intercalation of CO_3_^2-^ [22] derived from CO_2_ gas. The initial pH value of 12.3 was lowered to 6.8 by CO_2_ sparging. We expected hydrotalcite to increase the F fixation capacity in the neutral pH range; however, the fixation rate did not increase. This is due to the corruption of hydrotalcite, as discussed in the next section.

#### 3.2.4. Phase Identification of Regenerated Li Salt

**Figure 9:**
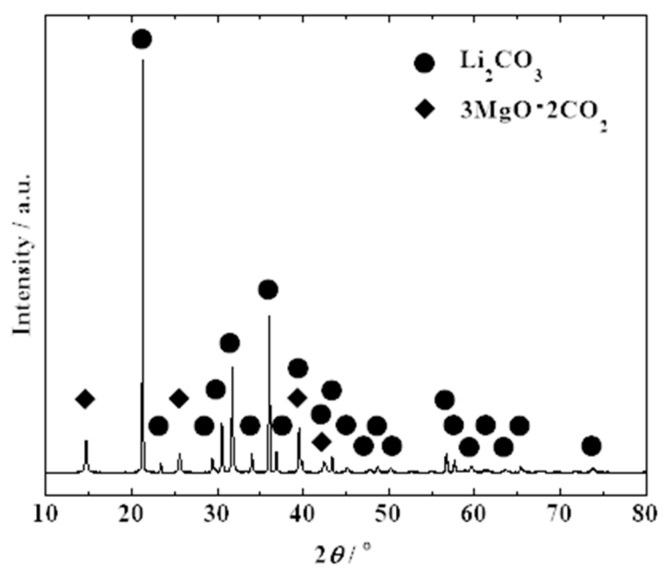
XRD profile of the regenerated Li salt.

**Figure 10:**
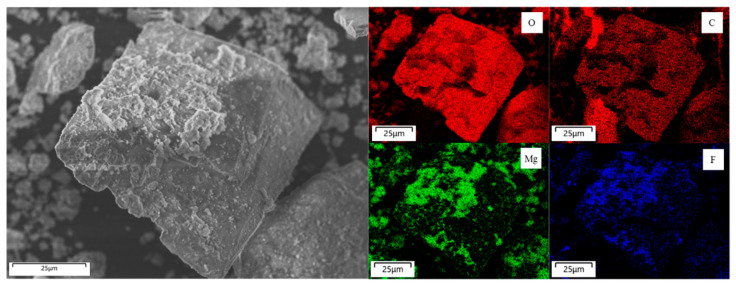
SEM images and elemental mapping of the regenerated Li salt.

Figure 9 shows the XRD profile of the regenerated Li salt obtained via multistep processes using Ca(OH)_2_ and hydrotalcite. Most of the diffraction peaks were identified as Li_2_CO_3_, and a few weak peaks were attributed to 3MgO·2CO_2_ [23]. Diffraction peaks of F-containing phases were not observed, which supports the advanced removal of F. Figure 10 shows SEM images and elemental mapping of the regenerated Li salt. Although Li in the salt state could not be observed because of its inability to produce characteristic X-rays, O and C were detected in the elemental mapping. Therefore, it can be assumed that Li_2_CO_3_ is the main component. In contrast, Mg derived from hydrotalcite was detected and the positions where F was present were identical. No diffraction peaks of MgF_2_ were observed in the XRD profile; however, this indicates the presence of Mg compounds as impurities. Considering that no Mg was detected in the aqueous solution during the F fixation test (Section 3.2.2), where only hydrotalcite was added, and that the SEM-EDS measurements of the regenerated Li salt did not confirm the presence of Ca, it was likely that an exchange reaction occurred between the dissolved Ca in the solution and the Mg in the hydrotalcite. Owing to the low solubility of the impurities such as CaCO_3_ and MgF_2_, they can be effectively separated by filtration during the concentration step in the purification of the regenerated Li salt. Sotiles et al. [24] reported exchange reactions of alkaline cations in LDHs. Because LDHs have a wide range of compositions, we believe that further modification of LDHs can suppress the undesired leaching of metals.

## 4. Conclusions

Herein, model samples of LiCoO_2_ mixed with PVDF were calcined at temperatures in the range of 350–700 °C under an Ar or air atmosphere to efficiently recover Li. Li was completely recovered by calcining the model sample at 400 °C under an Ar atmosphere, followed by water leaching.

To fix F from PVDF—an impurity in the purification of Li_2_CO_3_—Ca compounds (Ca(OH)_2_ and CaCO_3_) and hydrotalcite were used in dry and wet processes. In the wet treatment, F fixation was performed by adding Ca(OH)_2_ to a LiF solution with a F concentration of 1000 ppm. We found that 98.6% of F could be fixed by the two-process addition of Ca(OH)_2_, and residual Ca could be removed as CaCO_3_ by concentrating the solution. Hydrotalcite is also effective at immobilizing lower concentrations of F. In particular, the initial concentration of 25 ppm was reduced to 1 ppm, which is much lower than the lower limit (8 ppm) for the addition of Ca(OH)_2_.

The regenerated Li salt was identified as Li_2_CO_3_ with trace amounts of Mg. According to the SEM analysis, MgF_2_ impurities were observed in the regenerated Li salt, which may be due to the exchange reaction between Mg in the hydrotalcite and Ca dissolved in the aqueous solution.

## Figures and Tables

**Table 1:** Li/F ratio of the leaching solution obtained at different calcination conditions.

Calcination Temperature/°C	Li/F Ratio/Atomic Ratio	
	Ar	Air
350	1.8	-
400	3.2	2.3
450	3.2	-
500	3.1	1.1
600	3.4	1.1
700	3.6	-

**Table 2:** Change in total F fixation rate and Ca concentration at each process.

	Total F Fixation Rate/%	Ca Concentration/ppm
Primary process	98.2	57.4
After concentration	-	1.1
Secondary process	98.6	8.9

**Table 3:** F fixation rate and F concentration after secondary processing using calcium hydroxide and hydrotalcite.

Additive	Total F Fixation Rate /%	F Concentration /ppm	Initial pH /-	Final pH /-
Calcium hydroxide	89.1	109.5	7.8	12.3
Hydrotalcite	95.8	61.3	6.8	9.0

## Data Availability

The data can be provided by the corresponding author upon request due to participant privacy and confidentiality concerns.
